# Disease-related mutations predicted to impact protein function

**DOI:** 10.1186/1471-2164-13-S4-S11

**Published:** 2012-06-18

**Authors:** Christian Schaefer, Yana Bromberg, Dominik Achten, Burkhard Rost

**Affiliations:** 1 TUM, Bioinformatics - i12, Informatics, Boltzmannstrasse 3, 85748 Garching/Munich, Germany; 2 TUM Graduate School of Information Science in Health (GSISH), Boltzmannstr. 11, 85748 Garching/Munich, Germany; 3 Institute of Advanced Study (TUM-IAS), Lichtenbergstr. 2a, 85748 Garching/Munich, Germany; 4 Columbia University, Department of Biochemistry and Molecular Biophysics & New York Consortium on Membrane Protein Structure (NYCOMPS), 701 West, 168th Street, New York, NY 10032, USA; 5 Department of Biochemistry and Microbiology, School of Environmental and Biological Sciences, Rutgers University, New Brunswick, NJ 08901, USA

## Abstract

**Background:**

Non-synonymous single nucleotide polymorphisms (nsSNPs) alter the protein sequence and can cause disease. The impact has been described by reliable experiments for relatively few mutations. Here, we study predictions for functional impact of disease-annotated mutations from OMIM, PMD and Swiss-Prot and of variants not linked to disease.

**Results:**

Most disease-causing mutations were predicted to impact protein function. More surprisingly, the raw predictions scores for disease-causing mutations were higher than the scores for the function-altering data set originally used for developing the prediction method (here SNAP). We might expect that diseases are caused by change-of-function mutations. However, it is surprising how well prediction methods developed for different purposes identify this link. Conversely, our predictions suggest that the set of nsSNPs not currently linked to diseases contains very few strong disease associations to be discovered.

**Conclusions:**

Firstly, annotations of disease-causing nsSNPs are on average so reliable that they can be used as proxies for functional impact. Secondly, disease-causing nsSNPs can be identified very well by methods that predict the impact of mutations on protein function. This implies that the existing prediction methods provide a very good means of choosing a set of suspect SNPs relevant for disease.

## Background

### Evolution leads to genetic diversity

The selection of survival under changing conditions guides the cell’s genetic makeup (“genotype”) that is dynamically fit for retaining important cellular functions (“phenotype”). Today’s genetic landscape represents the current state of a sampling process that continuously creates new phenotypes. This process yields genetic variation across and within species. In human, single nucleotide polymorphisms (SNPs) are essential for genetic diversity [1,2]. Non-synonymous SNPs (nsSNPs) alter the amino acid sequence. Some of these mutations affect protein structure and/or function and could increase susceptibility to disease.

### Do disease-causing mutations impact protein function?

Disease-causing mutations occur often inside the protein (buried) and at hydrogen-bonding residues [3-5]. Protein function is often associated with evolutionarily conserved residues [4,6-9]. Most known disease-related nsSNPs in proteins of known 3D (three-dimensional) structure appear to affect structurally important residues and sites relevant for function [4]. For instance, disease-associated mutations can affect protein interactions [10]. In protein kinases, they have been shown to cluster into the functionally important catalytic core [11,12]. The above trends confirm the expectation that mutations cause disease because they damage important proteins.

Experts have established the above trends by laboriously inspecting small sets of well-curated proteins. Could less well-versed experts with better algorithms have established valid trends about disease-causing mutations for large data set by automatically extracting data set of disease-related mutations and their *predicted* functional effects? At OMIM’s infancy, a few years ago, we failed to accomplish this; i.e. observed trends did not differ much from random. This has changed. Here, we provide data that strongly suggest an affirmative answer to the question and demonstrate that we have a large repository of disease-causing mutations. To pick the most important practical result of our work: today’s disease-causing mutations can serve as an excellent proxy for “change of function”.

## Methods

### Data sets

We used SNPdbe [13] as the underlying source for amino acid substitutions, functional effect annotations and disease relations. This comprehensive new resource integrates variants from dbSNP [14], Swiss-Prot [15], PMD [16], and OMIM [17] and annotations of functional effects (from Swiss-Prot and PMD) and disease (from SwissVar [18], PMD and OMIM). The term ‘genetic disease’ is rather heterogeneous, covering Mendelian, monogenic disorders and polygenic diseases, exhibiting more complex genotypic patterns. Here, we do not differentiate between the different disease-types. Instead we aim at analyzing all disease-causing mutations.

We created the following five subsets from SNPdbe (Additional file 2). (1) *Set of disease-related *+* observed effect mutations:* We collected 1,105 human nsSNPs (from 217 proteins) that were annotated to be both disease-causing and functionally non-neutral. (2) *Set of disease-related mutations:* We obtained a set of amino acid substitutions in human proteins with disease-association. We extracted 26,404 mutations (3,419 proteins) with disease annotations but no annotated functional effect. (3) *Set of observed effect mutations:* We collected 36,317 mutants in 3,790 proteins with experimentally observed effect. We excluded mutations with disease associations. This set constitutes a part of the “functional effects” sets annotated in PMD; it served as the positive training set for SNAP [19]. Note that after our filtering the resulting set of mutations with *observed effect* and the set of *disease-related* mutants did NOT overlap. (*4*) *Set of mutations with unknown disease relation:* We extracted 251,414 variants (28,913 proteins) without known disease associations. (5) *Set of random mutations:* We randomly selected one mutation in each of the 28,913 proteins from the set of mutants of *unknown disease relation* such that the mutated position was maximally distant from any other mutation observed in the given protein.

### Prediction of effect

For the vast majority of point mutants (single amino acid changes or nsSNPs) in human, the impact on protein function remains unknown. For all mutations in the above four data sets (disease-causing, disease-relation unknown, observed function-changing, and random), we predicted their effects on function with SNAP [19] and SIFT [20]. Both methods provide binary classifications (effect/neutral) along with a more detailed score. SNAP scores range from -100 (strongly predicted as neutral) to 100 (strongly predicted to change function); the distance from the binary decision boundary (0) measures the reliability of the effect. Essentially, stronger predictions are also more reliable, *i.e.* the higher the score, the more likely the mutation impacts function [19,21,22]. For a small data set, we previously established that SNAP scores correlate with the severity of change; *i.e.* high (positive) SNAP scores relate to more severe functional effects [19,21,22].

SIFT [20] scores range from 0 to 1 and aim at characterizing the normalized probability of tolerable amino acid substitution. Values <0.05 imply prediction of functional change; all other values are considered neutral. As with many other prediction methods, the distance to the decision boundary (0.05) reflects the reliability of a particular prediction [23]. For many prediction methods developed in our group (protein-protein binding [24-26], protein-DNA binding [27], backbone flexibility [28]), the strength of an effect correlated with prediction strength, *e.g.* ISIS predicted binding hot spots stronger than other residues involved in the interaction [26]. Although we never used the strength of an effect to train our methods, this correlation is intuitive: stronger effects are more consistent and therefore become stronger carved into the machine-learning model. Similarly, SIFT scores could be used to prioritize amino acid substitutions [23]. In this perspective, we consider the distance from the default decision boundary (0.05) as the magnitude of the effect**.**

SNAP and SIFT aspire to solve the same problem with different means. SNAP was trained on literature-derived [16] mutants that are either functionally similar to the wild-type (neutral) or alter function (effect) in either direction (*decrease* and *increase* of function). SIFT on the other hand infers probabilities of functional change from residue conservation in alignments of evolutionarily related proteins. While SNAP operates on an experimentally substantiated definition of change, SIFT uses conservation scores of amino acids as a proxy for functional change. Although both methods largely capture the underlying biological meaning of functional change, their predictions disagree often. Thus, the methods are likely orthogonal, picking up different aspects of protein function.

In addition, we applied PhD-SNP [29] to predict whether mutations in all five sets are disease-causing or neutral. PhD-SNP offers several modes striking different balances between runtime and performance. We used the most accurate mode that uses both sequence and evolutionary profiles.

### Box plots

We represented our resulting distributions using box plots [30,31]. The lower and upper box edges depict the first and third quartiles of the distributions, respectively. The length of the box is the interquartile range of the distribution. The bold bar inside the box represents the median, while dashed lines reach to the most extreme data points, that are no more than 1.5 times the interquartile range away from the upper or lower box edge. Note that each box covers half the distribution.

## Results and discussion

### Disease-causing mutations strongly predicted to change protein function

**Figure 1:**
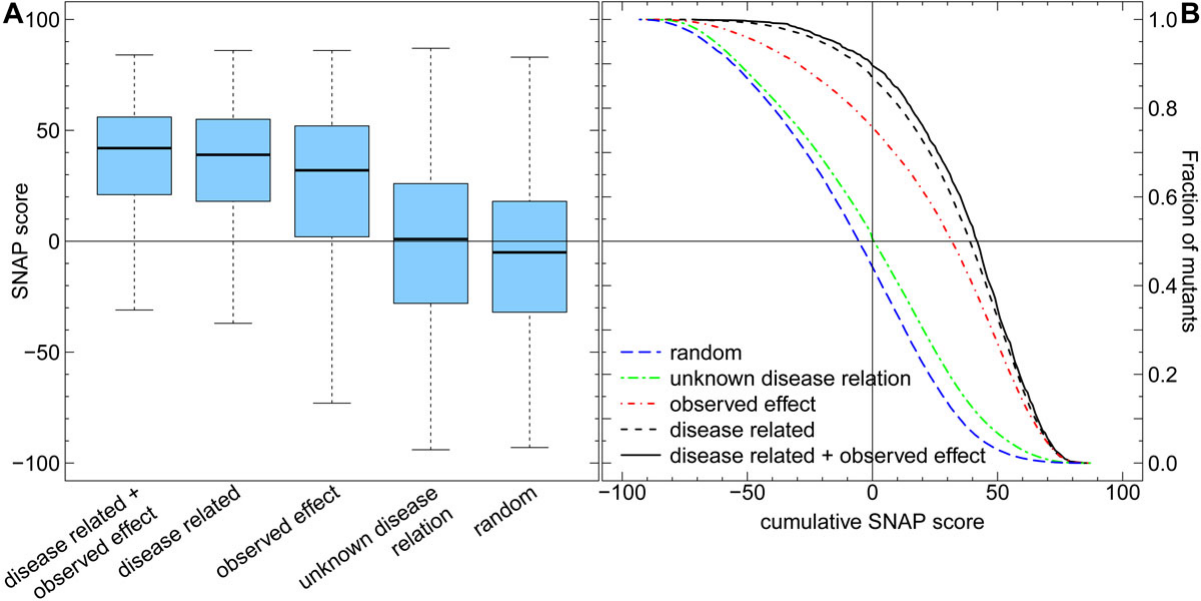
**Disease-causing mutations have highest scores** SNAP predicted the impact of function for five different data sets of point mutations: *disease related* + *observed effect* and *disease related* mutants, mutations with *observed effect*, *unknown disease relation*, and *random* mutations. For each set we display the predicted functional severity of mutations. (A) Scores above zero (horizontal line) correspond to *effect*, scores below to *neutral*, the distance from 0 correlates to severity; lower/upper bound and bar in the box represent the lower/upper quartile and median. 90% of *disease related*+*observed effect* and over 86% of the *disease related* mutations were predicted to effect function, compared to only 51% in mutations of *unknown disease relation*. Effect predictions dominated the *observed effect* mutants less (76%) than the *disease related* mutants (86%). The effect in *random* mutations (44%) provided an upper bound for effect mutations in proven non-disease related variants. (B) Cumulative distributions of predicted functional severity; points on a curve correspond to fractions (y-axis) of mutations with SNAP scores (x-axis) ≥ this value. The vertical line separates *neutral* from *effect*. Disease-causing mutations were predicted to be most severe (black solid and dashed lines above all others). These results suggest that change in function may explain most disease-related mutations.

**Figure 2:**
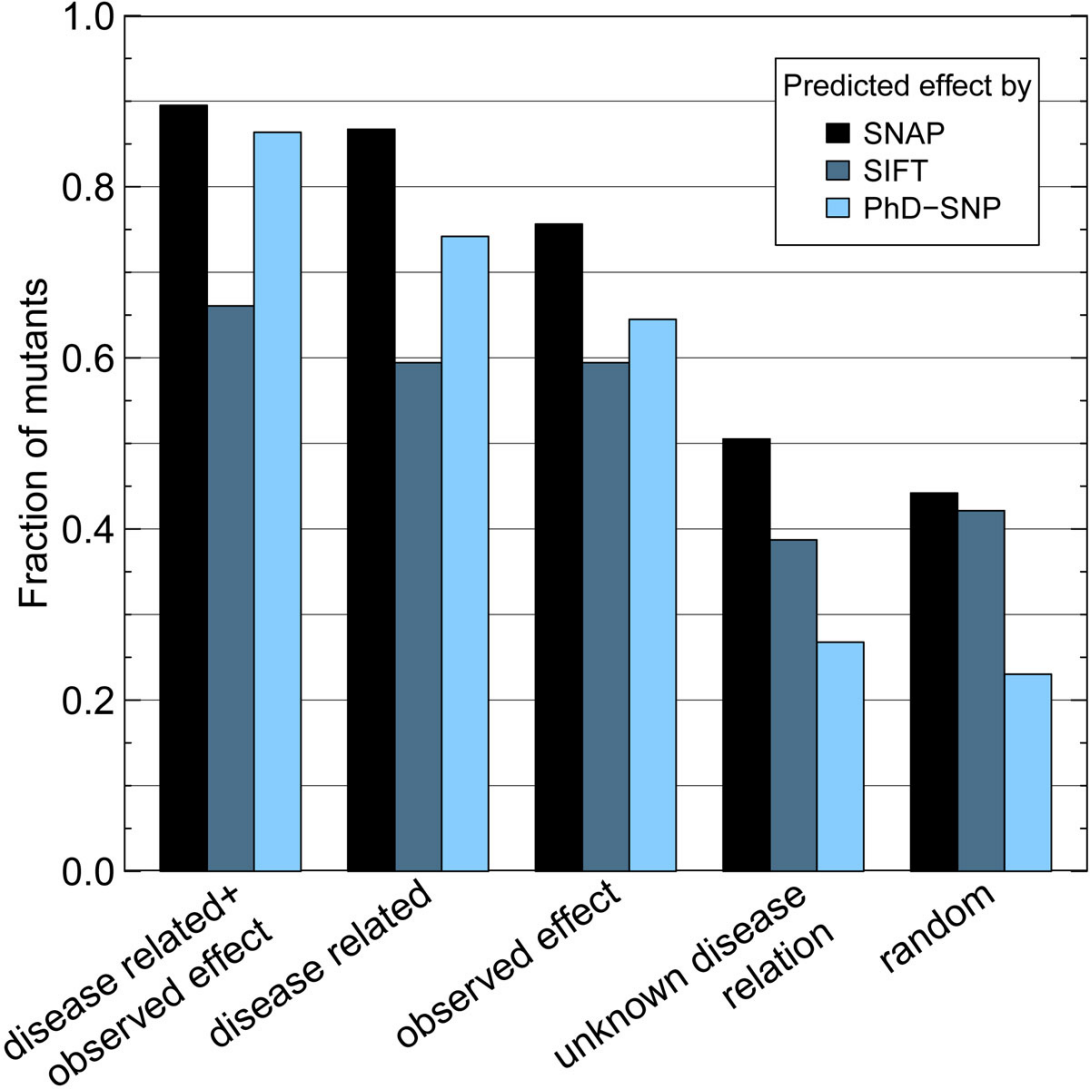
**Effect most prevalent in disease mutants** For each set we show the fraction of mutants with predicted effect (SNAP, SIFT: functional effect, PhD-SNP: disease). Disease predictions taken from PhD-SNP (light blue bars) confirm the major observation found in functional predictions (black+dark blue bars): *observed effect* mutants have high impact on disease. More than 64% of these are predicted to be disease-causing while only 27% of mutations of *unknown disease relation* are predicted to cause disease.

We applied SNAP and SIFT to the 26,404 annotated *disease related* mutants (Methods). At the default threshold, SNAP predicted over 86% of the *disease related* mutations to impact function (Fig. 1A, B, 2) and SIFT ~59% (Fig. 2, Additional file 1). SNAP predictions were very strong: about half of the effect predictions had levels of severity of >40 (Fig. 1B, dashed black curve).

In our experience, SNAP scores >40 are exceptional when applying the method to new data. To clarify this point, the *observed effect* mutations were the very same data set that trained SNAP. We ascertained that this set had no overlap with the *disease related* mutations (Methods). Usually, machine-learning methods perform much better on the training than on the testing set. This also holds for SNAP; hence, the distribution of SNAP scores for the training set of *observed effect* mutants is expected to be closer to ‘more effect’ than for any other data set. We observed the opposite (Fig. 1B: red vs. dashed black lines): effect predictions were stronger for the *disease related* mutations than for our *observed effect* training set, *e.g.* while just over 40% of the training set reached a score >40, 47% of the *disease related* mutations did. A difference of seven percentage points might not be perceived as high, but the effect is significantly higher for comparison to testing on the training set. SIFT overall also predicted the *disease related* mutations stronger than the *observed effect* data, but the difference was not significant (Additional file 1).

Do disease-related mutations *with* an observed effect alter function even more? We analyzed the predicted functional effect of disease-associated mutations *with* observed effect (*disease-related*+*observed effect*). About 90% were predicted to impact function (4% more than for *disease related*), while over 53% had SNAP scores higher than 40 (6% more than for *disease related*; Fig. 1A, B solid black line, Fig. 2). SIFT showed a similar trend: 66% in the set of *disease related*+*observed effect* compared to 59% in *disease related* mutations (Fig. 2, Additional file 1). This suggests that the most reliable source of impact mutations is by connecting disease relations and independent experimental observations.

As negative control, the predictions differed greatly for the 251,414 mutants with *unknown disease relation*. First, only about 51% of those were predicted to have an effect by SNAP (Fig. 1A, B, 2), and only 39% by SIFT (Fig. 2, Additional file 1). Second, only 12% of those had a SNAP score larger than 40 (Fig. 1B, dashed green curve).

### Many mutations with unknown effect predicted to alter function

SNAP and SIFT predicted much more effect for *disease related* mutations than in mutants with *unknown disease relation*. Still, many of those mutations were predicted to change protein function. However, much fewer mutants with *unknown disease relation* were predicted to significantly change function than the *disease related* mutations (Fig. 1B: strong effect for 14% of mutants *unknown disease relation* - dashed green line - vs. 48% of *disease related* mutations - dashed black line). Comparing the prediction trends between the two data sets suggests that the mutations of *unknown disease relation* will never become a ‘disease-rich’ set (i.e. through newly discovered disease associations). R*andom* mutations were even less often predicted to have strong effect (~7%, Fig. 1B, dashed blue line). This result suggests that many experimental annotations of ‘functional impact’ remain to be determined/observed for the set of mutations with *unknown disease relation* (roughly > 7%-14%).

### Same trend found in predicted disease mutations

If *disease related* can serve as a good proxy for (strong) functional impact, then a method trained to predict disease-causing mutations should reveal the reverse and thus confirm the same: predicted disease is expected to be enriched in *observed effect* compared to mutations of *unknown disease relation*. We analyzed the fraction of predicted disease by applying PhD-SNP (Methods) to our five data sets. PhD-SNP predicted >64% of the o*bserved effect* mutations as disease related (Fig. 2), while only 26% of mutations with *unknown disease relation* were predicted to be disease associated. Furthermore, we confirmed the other observations already found in functional impact predictions: Random mutations appear to have the lowest impact on disease (only 22%, Fig. 2).

PhD-SNP predicted both disease-related sets to contain most disease mutants (86% in *disease related*+*observed effect* and 74% in *disease related*, Fig. 2). This was expected due to the important overlap between our data and the training set of PhD-SNP [29]. Nonetheless, the increase in predicted-disease mutations of 12% once again suggested that *observed effect* mutants play a major role in disease.

Our findings show that if a mutation leads to disease then a change in function plays a major role in explaining the cause (59%-86%). This finding cannot be inverted due to the overlap of score distributions of *disease related* mutants and mutants with *unknown disease annotation* (Fig. 1A, Additional file 1); i.e. strong effect on function does not imply disease.

Our comparison between mutations annotated as *disease related* and those experimentally annotated function changing (*observed effect*) does not imply that there is anything special about disease-causing mutations. Instead, our findings highlight differences in the *severity* of functional effect. That is, on average, assuming that a disease causing mutation has a functional effect is more reliable than experimentally evaluating functional change.

## Conclusions

We compared disease-associated single point mutations (nsSNPs) predicted to change protein function with those of unknown disease-association. Implicitly, we tested the reliability of annotations that link mutations to disease and the extent to which predictions of functional effect overlap with disease causation.

As opposed to other studies addressing this question [3-6,10-12], we used predictions of functional effect to determine the fraction of deleterious point mutations in two different populations of human variants: *disease related* (or disease-causing) mutations and mutations without any knowledge of phenotypic effect. The major findings were: (1) annotations of disease-causation provide a good approximation of functional effect. (2) Methods developed to predict the impact of mutations onto protein function clearly identify disease-causing mutations as those that change function. In other words, their predictions provide a valuable first step towards the study of the molecular impact of disease.

## Authors' contributions

CS participated in the design of the study, performed the data analysis and helped to draft the manuscript. YB participated in the design of the study and helped draft the manuscript. DA participated in the design of the study. BR participated in the coordination and design of the study and helped to draft the manuscript.

## Competing interests

The authors declare they have no competing interests.

## Funding

This work was supported by a grant from the Alexander von Humboldt foundation through the German Ministry for Research and Education (BMBF: Bundesministerium fuer Bildung und Forschung); YB was supported by the SEBS, Rutgers, New Brunswick startup funds.

## Supplementary Material

Additional file 1 **SIFT predictions.** Non-neutral mutations are enriched in a set of disease-causing variants, whereas they are depleted in variants with no known linkage to disease. Click here for file

Additional file 2 **Mutation and sequence data.** Archive of the five different mutant sets used in this study separated by SNAP/SIFT and PhD-SNP predictions including the protein wild type sequences. Click here for file
