# Supplementary figures and images for: Disease-related mutations predicted to impact protein function
Source: BMC Genomics. 2012 Jun 18;13(Suppl 4):S11. doi: 10.1186/1471-2164-13-S4-S11 (PMC3394413; doi:10.1186/1471-2164-13-S4-S11)

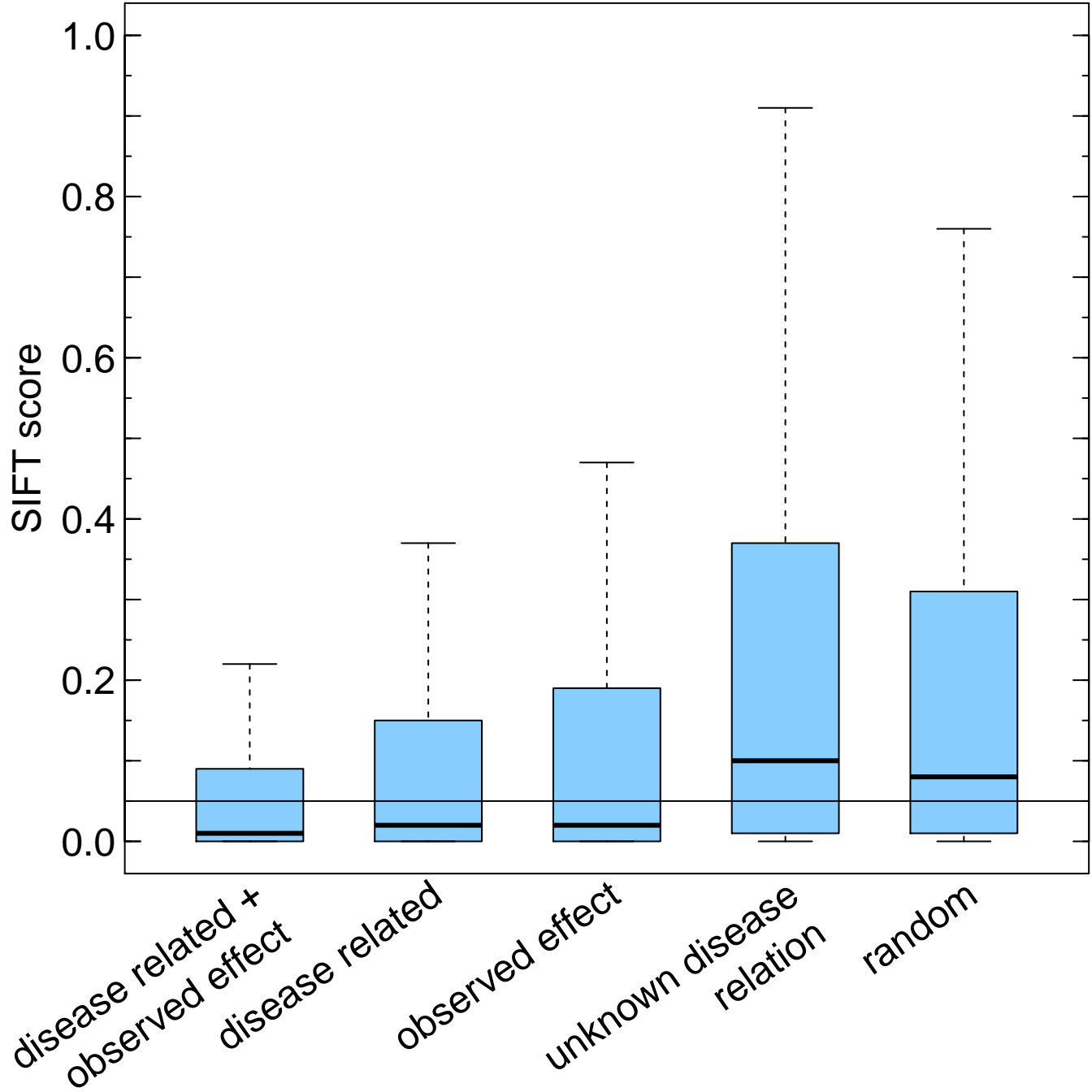

Supplement: Additional file 1 — SIFT predictions. Non-neutral mutations are enriched in a set of disease-causing variants, whereas they are depleted in variants with no known linkage to disease. [file 1471-2164-13-S4-S11-S1.pdf]
